# Supplementary figures and images for: Transcriptome and Proteomic Analysis Reveals Up-Regulation of Innate Immunity-Related Genes Expression in Caprine Herpesvirus 1 Infected Madin Darby Bovine Kidney Cells
Source: Viruses. 2021 Jul 2;13(7):1293. doi: 10.3390/v13071293 (PMC8310103; doi:10.3390/v13071293)

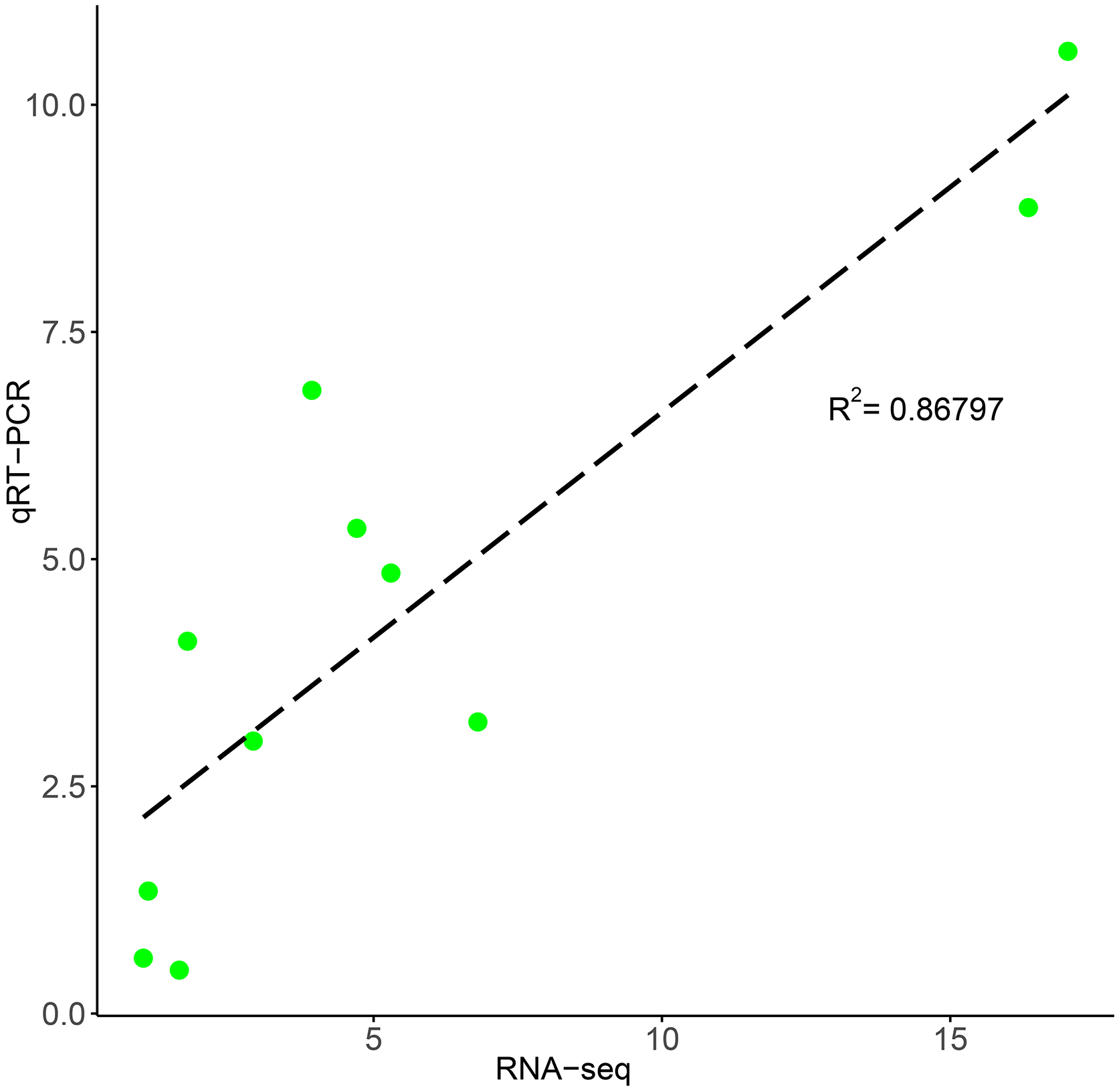

Supplement: Supplementary file 1 [file viruses-13-01293-s001.zip › Figure S1.tif]
